# Early Retinal Changes by OCT Angiography and Multifocal Electroretinography in Diabetes

**DOI:** 10.3390/jcm9113514

**Published:** 2020-10-30

**Authors:** Luisa Frizziero, Giulia Midena, Evelyn Longhin, Marianna Berton, Tommaso Torresin, Raffaele Parrozzani, Elisabetta Pilotto

**Affiliations:** 1 IRCCS—Fondazione Bietti, 00198 Rome, Italy; 2 Institute of Ophthalmology, Policlinico Gemelli, IRCCS, 00168 Rome, Italy; giulia.midena@gmail.com; 3 Department of Ophthalmology, University of Padova, 35128 Padova, Italy; longhin.evelyn@gmail.com (E.L.); tommaso.torresin@gmail.com (T.T.); raffaele.parrozani@unipd.it (R.P.); elisabetta.pilotto@unipd.it (E.P.); 4 ASST Rhodense, Garbagnate Milanese, 20024 Milan, Italy; bertonmarianna@libero.it

**Keywords:** diabetic retinopathy, multifocal electroretinography, optical coherence tomography, optical coherence tomography angiography, microaneurysms, early stages, morpho-functional correlation, vascular plexuses, hyperreflective intraretinal spots, retinal layers

## Abstract

Background: To evaluate the earliest retinal morphological and functional changes in diabetic eyes without or with early signs of diabetic retinopathy (DR). Methods: Twenty-two eyes with no DR (noDR group), 22 eyes with mild DR (DR group), and 18 healthy nondiabetic eyes (controls) were enrolled. All eyes were studied by means of spectral domain optical coherence tomography (OCT), OCT angiography (OCTA), and multifocal electroretinogram (mfERG). Results: A significantly higher number of OCT hyperreflective intraretinal foci (HRF) was found in both noDR and DR groups versus controls, but not between DR groups. The OCTA parameters of the superficial vascular plexus (SVP) were significantly reduced in the noDR group both versus controls and DR group (*p* < 0.05). The OCTA parameters of the intermediate capillary plexus (ICP) were significantly reduced in the DR group versus controls. An increased number of altered hexagons on mfERG was found in the noDR versus the DR group (*p* = 0.0192). Conclusions: Retinal vascular and functional parameters are differently involved in diabetic eyes; major vascular changes in the SVP and functional alterations of the mfERG are present in diabetic eyes with no clinical microvascular signs of DR, while ICP is mainly involved when early ophthalmoscopic signs of DR are present. The integrated use of mfERG and OCTA provides new significant insights into the pathogenesis of diabetic related retinal disease.

## 1. Introduction

Diabetic retinopathy (DR) has long been considered as a pure microvascular disorder; endothelial cell damage, pericyte loss, and secondary breakdown of the inner blood retinal barrier were thought to be the only drivers of the whole pathogenetic process [1]. With the development of optical coherence tomography angiography (OCTA) microvascular changes may be non-invasively visualized [2,3]. With OCTA, early distinct changes in the identifiable vascular retinal plexuses have been detected in diabetic eyes, even without clinically detectable DR [4,5,6]. However, both neurodegeneration and neuroinflammation have been more recently demonstrated to occur very early in diabetic eyes [7,8,9]. Diabetes mellitus (DM)-related damage induces a retinal neuroinflammatory response involving both the macroglial and the microglial cells [10]. It has been hypothesized that aggregates of activated microglial cells may be visualized in vivo at structural OCT as hyperreflective intraretinal foci (HRF), with specific features, in eyes with DM [11,12,13]. In diabetic eyes, the crosstalk among vessels, neurons, and glial cells in the retina is still partly unknown and we still lack a holistic approach which encompasses all retinal components [14,15]. A growing body of literature has been devoted to analyzing and interpreting both the new OCT and OCTA data [16,17]. However, few studies have tried to correlate the new morphological and vascular findings to macular functional parameters [18,19,20,21,22,23]. The aim of this study was to evaluate and correlate the earliest structural and vascular retinal changes—obtained by means of OCT and OCTA—and the electrophysiological functional parameters—measured by multifocal electroretinogram (mfERG)—in diabetic eyes without or with early signs of DR, compared to healthy controls. 

## 2. Experimental Section

This was an observational cross-sectional study with prospective enrolment. Diabetic patients with mild DR (DR group) according to the Clinical Diabetic Retinopathy Disease Severity Scale or without DR (noDR group), referred from November 2018 to February 2019, were consecutively recruited [24]. Age-matched healthy subjects were also enrolled, as control group. The main ocular exclusion criteria were: history of glaucoma or ocular hypertension, any intraocular disease other than DR, proliferative DR, the presence of diabetic macular edema (DME), refractive error >6 diopters, any intraocular surgery in the last six months, any concomitant topical treatment (except for artificial drops), a history of retinal laser treatment, and ocular media opacities precluding the quality of retinal imaging. The main systemic exclusion criteria were: the presence of any neurodegenerative disease or nervous system disorders unrelated to diabetes, any uncontrolled systemic diseases, and recent (3 months) glycated hemoglobin (Hba1c) ≥9%. All patients underwent a complete ophthalmic examination, including refraction and best-corrected distance visual acuity (BCVA) measurement, anterior segment examination, indirect ophthalmoscopy, and 90-D lens biomicroscopy. They also underwent spectral domain OCT, OCTA, and mfERG. BCVA measurement was performed in each eye by a certified examiner using standard Early Treatment Diabetic Retinopathy Study (ETDRS) protocol at 4 m distance with a modified ETDRS distance chart illuminator (Precise vision, Bloomington, IL, USA). An informed consent was obtained from each patient and the research was carried out in accordance with the Declaration of Helsinki.

### 2.1. Optical Coherence Tomography

Optical coherence tomography was performed using Spectralis HRA + OCT2 (Heidelberg Engineering, Heidelberg, Germany). This device is a confocal scanning laser ophthalmoscope with 870 nm wavelength and fundus infrared image as a reference. A TruTrackTM Active Eye Tracking system and automated real time (ART) modality allowed the elimination of motion artefacts. Two OCT scan patterns were performed for each eye: volume macular map (20° × 20°, 6 × 6 mm area, centered onto the fovea, 40,000 A-scan/s speed made of 49 horizontal B-scans (512 × 456 pixel) in ART modality (46 averaged images, 7 and 14 microns resolution, axially and laterally, respectively) and OCT linear scan (one 12 mm length scan centered onto the fovea at 180°, ART 100 averaged frames).

#### 2.1.1. Single Retinal Layer Thickness Measurements

An ETDRS grid centered onto the fovea subdivided the macular area in 9 parts, according to the incorporated Spectralis software, consisting of a central circular zone with a 1 mm diameter, and inner and outer rings of 3- and 6-mm diameter, respectively. The internal and external rings were subdivided into 4 quadrants. An automatic segmentation software (segmentation technology; Heidelberg Engineering, version 6.3.1.0) was used to identify and measure the thickness of retinal single layers. The algorithm detects 11 separation markers. For this study, the following retinal layers were considered: retinal nerve fiber layer (RNFL), ganglion cell layer (GCL), inner plexiform layer (IPL); inner nuclear layer (INL); outer plexiform layer (OPL); outer nuclear layer (ONL); outer retinal layer (ORL: external limiting membrane plus myoid zone, ellipsoid zone, and outer segments of the photoreceptors plus cone interdigitation with RPE and RPE/Bruch’s membrane complex). After automated segmentation, manual refinement was eventually performed in case of errors or artefacts. The mean thickness for each layer was considered as the mean value of the 4 quadrants of both inner and outer rings; the central 1 mm diameter ring was considered as the total mean thickness. 

#### 2.1.2. Hyperreflective Intraretinal Foci

**Figure 1:**
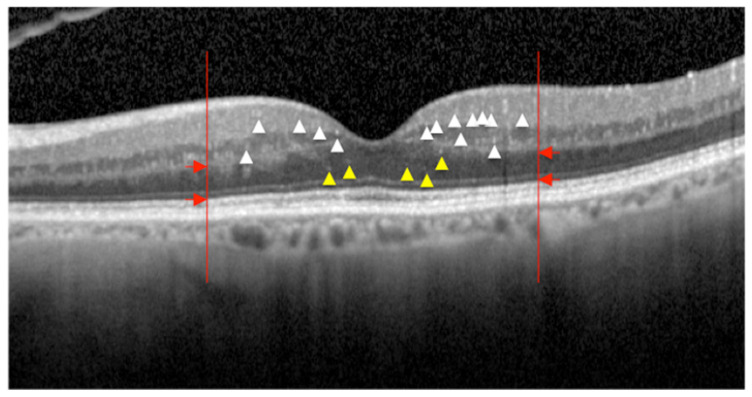
Linear spectral domain optical coherence tomography scan centered onto the fovea showing hyperreflective intraretinal foci (HRF) detection. The vertical red lines border the area where HRF are counted (3 mm length). The red arrows indicate the boundary between the inner retina (from the inner limiting membrane to the lower border of the inner plexiform layer) and the outer retina (from the upper limit of the outer nuclear layer and the boundary of the retinal pigment epithelium—Bruch’s membrane). White and yellow arrowheads indicate HRF in the inner and outer retina, respectively. Only HRF with small dimension (≤30 micron), intermediate reflectivity (similar to retinal nerve fiber layer) and no back-shadowing were considered.

HRF were counted in the central 3 mm length of the structural OCT linear scan both in the inner retina (IR), defined as all layers between the INL and the lower limit of OPL, and in the outer retina (OR), defined as the layers between the upper limit of the ONL and the RPE and the Bruch’s membrane (RPE/BM) boundary (Figure 1) [25]. 

Only HRF with specific, previously described characteristics (maximum diameter ≤30 μm, moderate reflectivity (similar to RNFL) and absence of back shadowing) were considered [11,25]. An experienced masked grader performed all OCT measurements.

### 2.2. Optical Coherence Tomography Angiography

All OCTA images were acquired by OCTA Spectralis HRA+OCT2 platform (Heidelberg engineering, Heidelberg, Germany), Heyex software 6.9a. The instrument has an A-scan rate of 85,000 scans/s and has a scanning laser confocal ophthalmoscope with 870 nm wavelength. TruTrackTM Active Eye Tracking and ART allow improving of the image quality. The signal amplitude-decorrelation algorithm by Heidelberg engineering is applied on a volumetric scan 10° × 10° (around 3 × 3 mm) made of 512 B-scan (512 × 512 pixels) in ART modality (7 averaged frames with axial resolution 3.9 μm and 5.7 μm). A B-scan angiogram is generated by the decorrelation of standard B-scans sequentially acquired on the transversal section, generating contrast between static and dynamic tissue. Automatic segmentation algorithm Heyex Software 6.9a is able to distinguish the superficial vascular plexus (SVP, from RNFL to IPL- [IPL-corresponds to 17 μm above IPL]), the intermediate capillary plexus (ICP, from IPL- to IPL + [IPL + corresponds to 22 μm below IPL]), and the deep capillary plexus (DCP, from IPL + to OPL) [2]. Thus, high quality en-face OCTA images were automatically provided. The in-built projection artifact removal (PAR) tool ensured the removal of artifacts using information from the superficial vascular plexus. OCTA images without sufficient quality for analysis, signal strength (SS) inferior to 30 in “Q score” (on a scale of 0 to 40 for Spectralis, Heidelberg, Germany) or with segmentation errors, were excluded. A skilled technician performed all scans and checked each image after acquisition to detect any motion artifact or segmentation errors and eventually repeated examination.

#### OCTA Quantitative Parameters

**Figure 2:**
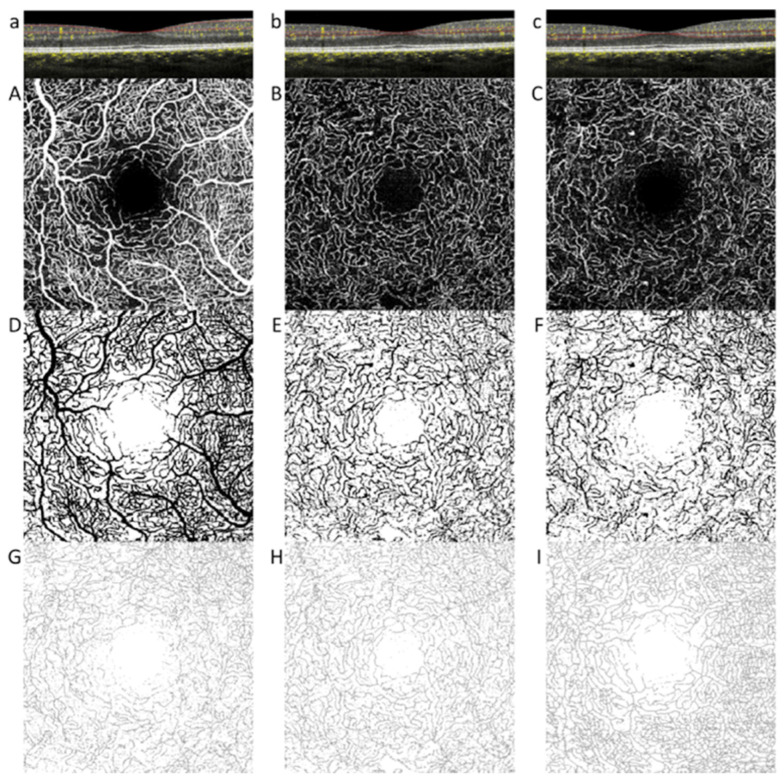
En-face optical coherence tomography (OCT) angiography of the superficial vascular plexus (SVP), intermediate (ICP) and deep capillary plexus (DCP) with the corresponding slabs at the OCT B scan (**a**–**c**). The SVP (**A**) en-face image was generated by a slab extending from RNFL to inner plexiform layer (IPL)-(IPL-corresponds to 17 μm over IPL), the ICP (**B**) by a slab extending from IPL− to IPL+ (IPL + corresponds to 22 μm below IPL) and the DCP (**C**) by a slab extending from IPL + to outer plexiform layer (OPL). Automatically binarized (**D**–**F**) and skeletonized (**G**–**I**) images of each plexus with ImageJ software.

The foveal avascular zone (FAZ) measurement was performed manually using the incorporated software tool. FAZ area was traced on the OCTA image of the SVP, ICP, and DCP, and automatically calculated in mm^2^. A quantitative analysis of the vascular plexuses at the OCTA images was performed using an open-source available ImageJ software (National Institutes of Health, Bethesda, MD, USA). The images with significant artefacts, precluding a correct examination, were excluded from the study. To quantify each bi-dimensional en-face image, four parameters were analyzed: vessel area density (VAD); vessel length fraction (VLF); vessel diameter index (VDI), and fractal dimension (FD) (Figure 2) [25]. 

Briefly, OCTA en-face images were automatically converted into binarized images on ImageJ. The VAD was obtained by dividing the number of black pixels counted by the software in the binarized image by the total number of image pixels. A skeletonized image was then automatically elaborated from the binarized image until a single pixel remains for each vessel segment. The VLF was calculated by dividing the number of vessels pixels obtained by the software in the skeletonized image by the total number of image pixels. The VDI was obtained by processing both the binarized and the skeletonized images to calculate the average vessel caliber. Finally, the FD is an index of complexity of the vessels’ ramification. It was obtained on a skeletonized image by a specific count of differently sized squares containing a vessel fragment (Figure 2).

### 2.3. Multifocal ERG

The mfERG recording was performed according to the International Society for Clinical Electrophysiology of Vision (ISCEV) standard using the Visual Evoked Response Imaging System (VERIS 6.3.2, Electro-Diagnostic Imaging Inc., Milpitas, CA, USA) [26]. After dilation of the pupils (1% tropicamide eye drops) and topical anesthesia (benoxinate hydrochloride 4 mg/mL eye drops) a corneal ERG-Jet (ERG-Jet, Fabrinal eye care, La Chaux de Fonds, Switzerland) electrode was placed on the corneal surface as active electrode; while the reference and ground skin electrodes were placed near the orbital rim temporally to the tested eye and on the forehead, respectively. The fellow eye was occluded. Impedance was always <5 kΩ. The stimulus pattern was displayed on a micro-display CCD (FMSIII stimulator), which allowed for manual correction of refractive spherical errors. Patients were asked to fixate on the red fixation target in the center of the pattern. The fixation was monitored using an eye camera. The stimulus consisted of 103 hexagonal elements scaled with eccentricity; each element was independently alternated between white (luminance of 200 cd/m^2^) and black (luminance of <1 cd/m^2^) at the frequency of 75 Hz according to a binary m-sequence. Each recording was subdivided into 19 short segments of 30 s each; if blinks or other movements produced excessive noise, the segment was rejected and repeated. The recorded signals were band pass filtered between 10 and 300 Hz and were amplified 50,000 times. 

#### mfERG Parameters

The 103 traces were grouped into 6 concentric rings from center to periphery (R1–R6). For each ring the implicit time (N1-IT, ms) and Amplitude (nV/deg^2^) of N1 wave (N1-A, measured from the isoelectric to the trough of N1), the A of P1 wave (P1-A, from trough of N1 to peak of P1), and the implicit time (P1-IT) were measured. By superimposition of the OCT ETDRS macular map over the mfERG hexagonal pattern, the single 19 hexagons included in the three central rings (R1, R2, R3), corresponding to macular area were additionally analyzed. Both P1-IT and P1-A were compared among groups (control, noDR and DR) for each hexagon. Moreover, the number of abnormal central hexagon was quantified: each hexagon was classified as abnormal when the z score was of 2 or higher for P1-IT and −2 or lower for A. An eye with a central mfERG abnormality was defined as having at least one abnormal hexagon in any of the three central rings [27]. A single masked operator performed all mfERG measurements. 

### 2.4. Statistical Analysis

Quantitative parameters were described as mean value and standard deviation; qualitative parameters were expressed as frequency distribution and relative percentage. The comparison among groups was conducted first by considering all diabetic eyes (All Diabetics group) versus controls (Control group) with analysis of variance (ANOVA) for repeated measurements; all measurements were replicated in both eyes of each patient. For the comparison of the different parameters among the distinct groups (noDR group; DR group and Control group) the variance analysis (ANOVA,) for repeated measures was first performed. Thereafter, the Bonferroni post-hoc test among groups was performed. The analyses were repeated on each group and T-student test on the regression coefficient significance was performed to verify the correlation among parameters. All the analyses were made using SAS^®^ 9.3 statistical software (SAS-Institute, Cary, NC, USA) on personal computer. *p*-value has been interpreted as statistically significant when <0.05 where not otherwise specified.

## 3. Results

Forty-four eyes (All Diabetic group) of 22 Caucasian diabetic patients—22 eyes of 11 patients with no DR (noDR group) and 22 eyes of 11 patients with mild DR (DR group)—and 18 eyes of nine healthy volunteers (control group) were included in this study. The best corrected visual acuity was 85 ETDRS score in all studied eyes. There were no statistically significant differences among groups for demographic parameters, and systemic diabetes characteristics between noDR and DR groups. The main characteristics of the study population are summarized in Table 1.

### 3.1. OCT Parameters

The mean thickness of all single retinal layers both in the central 1 and 6 mm of the macular area did not significantly differ among groups. However, RNFL, GCL, IPL, INL, and ONL were thinner and ORL was thicker in both diabetic groups compared to controls, mainly in the DR group (Table 2). 

**Figure 3:**
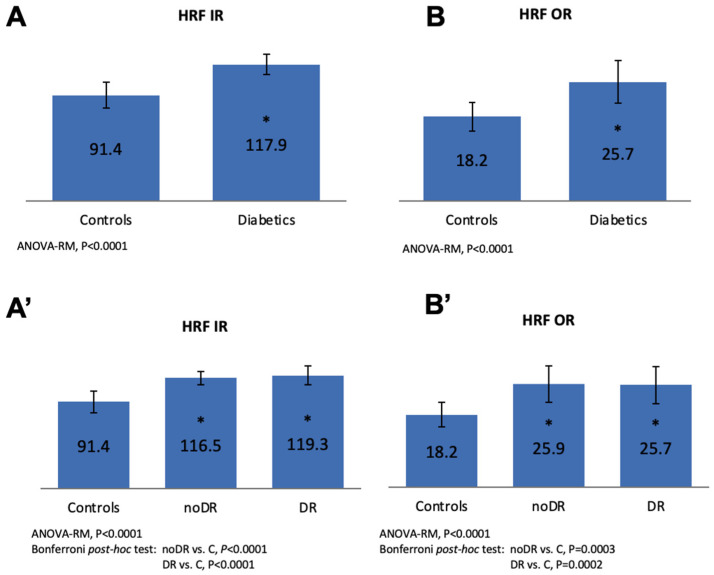
Representation of mean number of hyperreflective intraretinal foci (HRF) in the inner (IR) and outer retina (OR) in all diabetic eyes versus healthy controls (**A**,**B**) and in diabetic eyes without DR (noDr) and with mild DR (DR) versus controls (**A’**,**B’**). A significantly increased number of HRF in the IR and OR of all diabetics versus controls and in each diabetic group versus controls was found. * Statistically significant difference compared to controls.

The number of HRF was significantly higher, both in the inner and outer retina, in all diabetic group versus controls (both *p* < 0.0001), and in both noDR and DR groups versus controls (HRF in IR: *p* < 0.0001 for both; HRF in OR: *p* = 0.0003 for noDR vs controls and *p* = 0.0002 for DR vs controls). The number of HRF did not differ between noDR and DR group. (Figure 3).

### 3.2. OCTA Parameters

No significant difference was found in FAZ area of SVP, ICP, and DCP between all diabetic, noDR, and DR groups, versus controls.

**Figure 4:**
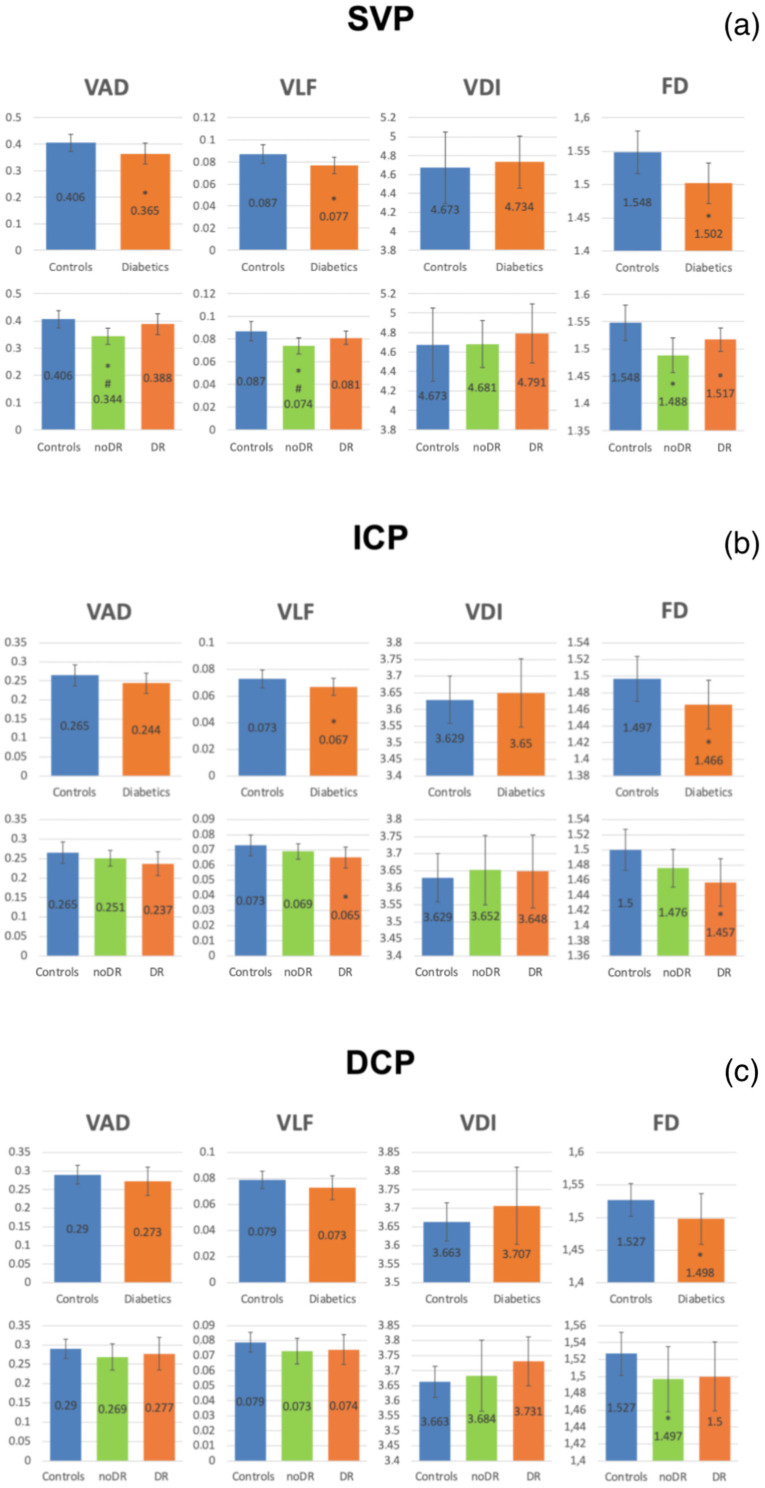
Representation of vessel area density (VAD), vessel length fraction (VLF), vessel diameter index (VDI), and fractal dimension (FD) of the superficial vascular plexus (SVP, **a**), intermediate (ICP, **b**) and deep capillary plexus (DCP, **c**) in all diabetic eyes versus controls (upper row) and in diabetic eyes without diabetic retinopathy DR (noDR) and with DR (DR), separately compared to control group (lower row). (**a**) significant reduction of VAD, VLF and FD was found in diabetic eyes versus controls. The VAD and VLF of noDR group showed a significant reduction compared to controls and to DR. FD showed a significant reduction in both noDR and DR groups versus controls. (**b**) significant reduction of VLF and FD was found in diabetic eyes versus controls and confirmed in DR group, when separately evaluated. (**c**) significant reduction of FD was found in diabetic eyes versus controls and still present in noDR group, when separately evaluated. * Statistically significant difference compared to controls; # statistically significant difference compared to DR group

In all diabetic groups, all OCTA vessels parameters of the SVP (VAD, VLF, and FD) and the VLF and FD of ICP, and FD of DCP were significantly reduced versus controls (*p* < 0.05). When considering the two diabetic groups separately (noDR and DR), the VAD, VLF, and FD of the SVP were reduced in the noDR group, not only versus controls (*p* = 0.0002 for all) but also versus the DR group (*p* = 0.0056 for VAD, and 0.0375 for VLF). At the ICP level, VLF and FD were both significantly reduced in the DR group versus controls (*p* = 0.0217 and 0.0067, respectively). At the DCP level, only FD was reduced in the noDR group versus controls (*p*-value = 0.0415). (Figure 4).

### 3.3. Multifocal ERG Parameters

The comparison of the six concentric rings (R1–R6) did not show any statistical difference among groups, considering P1-A, P1-IT, N1-A and N1-IT. 

**Figure 5:**
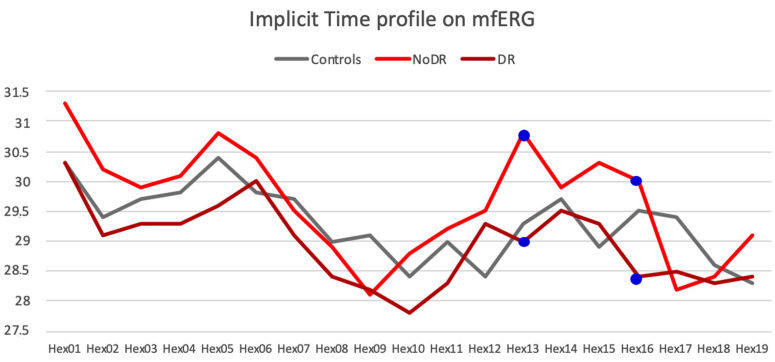
Representation of the implicit time (IT) on multifocal electroretinography (mfERG) waves in controls, no Diabetic Retinopathy (DR) and DR groups. In hexagon 13 and 16 there is a significantly increased IT in the noDR versus the DR group (*p* = 0.0273 and *p* = 0.0471, respectively).

However, the IT of the 19 central hexagons increased in the noDR versus the other groups, reaching statistical significance between the DR and the noDR group, in hexagon 13 and 16 (*p* = 0.0273 and *p* = 0.0471 respectively). (Figure 5).

Moreover, the analysis of the central hexagons showed a significantly greater number of altered hexagons in the noDR compared to the DR group (5.26 vs 2.11, *p* = 0.0192).

### 3.4. Correlations between Morphologic and Electrophysiologic Parameters

Correlations among OCTA parameters (VAD, VLF, VDI, FD) of the three retinal plexuses and the number of the HRF in the inner and outer retina were calculated. Across all searched correlations, the statistical significance was mostly sporadic and rarely coherent.

## 4. Discussion

Using an integrated retinal morphological and functional approach by means of OCT, OCTA, and mfERG, we detected that both structural and vascular retinal changes and functional alterations were present in diabetic eyes compared to healthy controls. However, when comparison was performed between eyes with or without DR, vascular retinal and functional retinal changes were differently present. 

With structural OCT, the presence of HRF in the inner and outer retina was evaluated. In recent years, inflammation received great attention in the pathophysiology of DR, and HRF are supposed to represent an in vivo OCT biomarker of retinal inflammation, as aggregates of activated microglial cells [11,12,28]. In the present study, HRF increased both in the inner and outer retina in diabetic eyes versus controls, with no differences between the diabetic groups, suggesting that local retinal inflammation occurs early, before clinically detectable retinopathy.

With OCT, the analysis of single retinal layers thickness showed a slight progressive thinning of the inner retina layers, specifically RNFL, GCL, IPL, and INL and a mild thickening of ORL in diabetic eyes compared to controls, even if a statistically significant difference was not reached. The thinning of inner retina has already been reported, as secondary to increased apoptotic phenomena of the retinal neuronal cells in DM [29,30]. Histopathologic studies have shown that ganglion cells dendrites are the first cellular component damaged in diabetic models, followed by their axons and cellular bodies [31,32]. Otherwise, the mild thickening of the ORL detected in the present study is difficult to clearly understand, as contrasting results have been reported in the literature, perhaps influenced by the different segmentation of the outer retina [29,33,34,35] However, morphologic changes of the photoreceptors (swelling of the inner segments and the disorganization or lack of outer segments) have been described in diabetic animal models. [34,35] Nonetheless, OCT thickness provides a general measure of the retinal integrity, involving both the neuronal and the non-neuronal elements, limiting, at these early stages, our understanding of the complex mechanisms leading to thickness changes [9,36]. OCTA provides a subtle and precise analysis of retinal flow, and allowed us to detect significant differences among different retinal plexuses, which may be related to the metabolic activity of the different components of the corresponding retinal layers [37].

OCTA analysis confirmed the presence of retinal vascular changes in diabetic eyes, even with mild or no DR, as previously reported [4,5,6]. However, when evaluating the two diabetic groups and the three single retinal vascular plexuses separately, distinct specific changes were found. In no-DR eyes, OCTA parameters (VAD, VLF, and FD) in the SVP were significantly reduced compared to both DR eyes and controls, with no changes in the other two deeper plexuses. Onishi et al. also found changes of SVP parameters on OCTA even in preclinical stages of DR, and concluded that this early alteration may help to distinguish healthy from diabetic eyes [38]. Moreover, we specifically found a significant macular functional alteration by mfERG in noDR, showing an early functional impairment more evident in patients without clinical signs of DR but with a rarefaction of SVP. A correlation between ERG parameters and the superficial vascular density has already been suggested by Kim et al. [39]. However, in that study the use of full field ERG, instead of mfERG, evaluated the electrical activity generated throughout the whole retina, whereas we studied the mfERG response, localized at the posterior pole, corresponding to the area of analysis of OCTA [39].

In the DR group we detected that the ICP was the only plexus with significant changes compared to both controls and the no-DR group. This group was represented by eyes affected by mild DR, thus mostly characterized by the presence of microaneurysms, which mainly originate in the INL, where the deeper retinal plexuses are located [40]. ICP has a peculiar auto-regulation, which can explain the early changes we detected [37,38,41]. It is localized in the inner part of the INL, where capillaries arise from retinal arterioles, and supplies the neurons of the inner retina, characterized by a high metabolic demand [37]. Therefore, ICP shows higher sensitivity to hypoxia and immediate flow response to metabolic demand, explaining histopathologic and OCT evidence that microaneurysms preferentially cluster in the INL in early DR, and OCTA detection of ICP vascular changes [5,37,41]. Moreover, at this level are also located the nuclei of Müller cells, which are activated early by hyperglicemia and are primarly involved in the homeostasis of the neurovascular unit. Therefore, their activation may contribute to the early changes detected at ICP in early (mild in the Clinical Diabetic Retinopathy Disease Severity Scale) DR eyes [42].

The general characteristics of our diabetic population (Table 1) were comparable in the no-DR and the DR groups. Therefore, our data seems to suggest the presence of different and specific morphological and functional characteristics that may characterize the two subgroups of this diabetic population without macular edema: in no-DR eyes major alterations in the SVP and functional changes at mfERG were detectable, whereas in DR eyes more changes of the deeper intraretinal plexuses, mainly in ICP, seemed evident. Santos et al. already noted in the EUROCONDOR study population, a discrepancy between patients with no apparent fundus abnormalities but mfERG alterations and patients with early microvascular impairment but without mfERG alterations, suggesting the presence of two different phenotypes, one more prone to develop microvascular alterations and the other characterized by main neurodegenerative abnormalities [27]. Our data confirms the presence of different vascular and functional characteristics in patients with and without ophthalmoscopic signs of DR, showing a different involvement of retinal plexuses at OCTA in these two types of patients.

This may strongly influence the prognosis for these patients and may explain the difficulties that ophthalmologists may sometimes face in the prognostic evaluation, particularly when just based on the International Diabetic Retinopathy and Macular Edema Severity Scale. This scale is mainly based on the vascular pathologic features detected at ophthalmoscopy, not considering other equally relevant, but more recently detected, components, such as diabetes-induced neurodegeneration [24]. Technological advances in ocular morphofunctional evaluation, as well as new discoveries in diabetic retinal neuropathy are beyond the current DR classifications. The need for an extended, comprehensive classification of diabetic retinal disease has already been proposed. [43] The individuation of different phenotypes of DR, different from the conventional DR severity grades, could be the first step towards a more exhaustive and individualized approach to DM patients, from a diagnostic but also prognostic and therapeutic perspective. Another hypothesis is that our data document the presence of an early (probably limited in time) effort of the retinal tissue to a morphologic and functional restoration after a precocious damage by chronic (often unknown) hyperglycemia. A long-term longitudinal follow-up of our patients is ongoing and might determine, with other prospective studies, the significance of these different behaviors [42].

The majority of studies on OCTA has considered only two retinal capillary plexuses (SVP and DCP), making any comparison difficult to be performed [44,45]. The more recently available software of OCTA devices can automatically detect the ICP, separately from the SVP, along which it was usually analyzed. Our findings confirm the importance of considering the two capillary plexuses separately in the evaluation of diabetic eyes. Moreover, the introduction of OCTA has expanded our ability to detect the microvascular retinal changes secondary to diabetes, showing that the evaluation of DM patients based only on ophthalmoscopy-detectable vascular changes, which affect the deeper capillary plexuses, may be significantly limiting.

In conclusion, this study confirms the multifactorial pathogenesis of DM-related damages, while the absence of specific correlations among morphological and functional parameters may suggest that vascular damage, neurodegeneration, and retinal inflammation independently contribute to the pathogenesis of the diabetic related retinal disease. The evaluation of specific morphological and functional parameters, using an integrated morphofunctional approach, seems to be an adequate way to detect the different DM-related retinal changes.

## Figures and Tables

**Table 1:** Demographic characteristics of the enrolled population.

	Control Group	noDR Group	DR Group
Eyes, n.	18	22	22
Age, years, mean ± SD	50.0 ± 14.8	51.9 ± 16.3	49.5 ±12.5
Sex (M:F)	4:5	6:5	6:5
Duration, years, mean ± SD	NA	14.5 ± 8.0	15.8 ± 9.3
Type of DM (1:2)	NA	3:8	4:7
IOP, mmHg, mean ± SD	15.8 ± 1.3	17.3 ± 1.9	16.2 ± 2.1
Refractive error, diopters, mean ± SD	−0.15 ± 1.31	−0.06 ± 1.17	−0.22 ± 0.87
BCVA, ETDRS score, mean ± SD	85 ± 0	85 ± 0	85 ± 0

For all parameters no statistically significant difference among groups was found. N: number; DR: diabetic retinopathy; SD: standard deviation; DM: diabetes mellitus; M: male; F: female; IOP: intraocular pressure; BCVA: best corrected visual acuity; ETDRS: Early Treatment Diabetic Retinopathy Study; NA: not applicable.

**Table 2:** Retinal layers thickness in the Early Treatment Diabetic Retinopathy Study (ETDRS) map (6 mm diameter).

	Controls	All Diabetics Group	*p*-Value *	noDR Group	DR Group	*p*-Value *
RNFL	29.4 ± 2.5	28.7 ± 2.5	0.5899	29.0 ± 3.2	28.5 ± 1.5	0.7090
GCL	43.5 ± 1.6	42.5 ± 2.4	0.1653	42.9 ± 2.5	42.1 ± 2.4	0.0574
IPL	35.2 ± 1.3	35.0 ± 2.0	0.6570	35.1 ± 2.2	34.9 ± 1.8	0.8156
INL	35.3 ± 1.6	34.8 ± 1.5	0.2358	34.9 ± 1.5	34.8 ± 1.6	0.4925
OPL	29.7 ± 2.2	30.4 ± 3.0	0.4610	29.4 ± 2.7	31.5 ± 3.0	0.1052
ONL	66.2 ± 6.0	63.4 ± 8.9	0.4078	64.3 ± 7.0	62.5 ± 10.5	0.6387
ORL	80.5 ± 2.8	84.0 ± 13.2	0.0845	82.5 ± 3.0	85.5 ± 18.5	0.1896

* ANOVA *p*-value of the comparison of each diabetic group to controls. All measurements are expressed in μm, as mean value ± standard deviation. ETDRS: Early Treatment Diabetic Retinopathy Study; DR: diabetic retinopathy; RNFL: retinal nerve fiber layer; GCL: ganglion cell layer; IPL: inner plexiform layer; INL: inner nuclear layer; OPL: outer plexiform layer; ONL: outer nuclear layer; ORL: outer retinal layers.
